# Learning to work together – lessons from a reflective analysis of a research project on public involvement

**DOI:** 10.1186/s40900-016-0051-x

**Published:** 2017-01-09

**Authors:** A. Howe, E. Mathie, D. Munday, M. Cowe, C. Goodman, J. Keenan, S. Kendall, F. Poland, S. Staniszewska, P. Wilson

**Affiliations:** 1 Norwich Medical School, University of East Anglia, Norwich, NR4 7TJ UK; 2 School of Health Sciences, University of East Anglia, Norwich, UK; 3 Centre for Health Services Studies, University of Kent, Canterbury, UK; 4 Centre for Primary and Community Care at the University of Hertfordshire, Hatfield, UK; 5 RCN Research Institute at Warwick Medical School, Coventry, UK

**Keywords:** Patient and public involvement in research, Partnership working, Stakeholder engagement

## Abstract

**Plain English summary:**

Patient and public involvement (PPI) in research is very important, and funders and the NHS all expect this to happen. What this means in practice, and how to make it really successful, is therefore an important research question. This article analyses the experience of a research team using PPI, and makes recommendations on strengthening PPI in research.

There were different PPI roles in our study – some people were part of the research team: some were on the advisory group; and there were patient groups who gave specific feedback on how to make research work better for their needs. We used minutes, other written documents, and structured individual and group reflections to learn from our own experiences over time.

The main findings were:- for researchers and those in a PPI role to work in partnership, project structures must allow flexibility and responsiveness to different people’s ideas and needs; a named link person can ensure support; PPI representatives need to feel fully included in the research; make clear what is expected for all roles; and ensure enough time and funding to allow meaningful involvement. Some roles brought more demands but also more rewards than others - highlighting that it is important that people giving up their time to help with research experience gains from doing so. Those contributing to PPI on a regular basis may want to learn new skills, rather than always doing the same things. Researchers and the public need to find ways to develop roles in PPI over time. We also found that, even for a team with expertise in PPI, there was a need both for understanding of different ways to contribute, and an evolving ‘normalisation’ of new ways of working together over time, which both enriched the process and the outputs.

**Abstract:**

**Background**

Patient and public involvement (PPI) is now an expectation of research funders, in the UK, but there is relatively little published literature on what this means in practice – nor is there much evaluative research about implementation and outputs. Policy literature endorses the need to include PPI representation at all stages of planning, performing and research dissemination, and recommends resource allocation to these roles; but details of how to make such inputs effective in practice are less common. While literature on power and participation informs the debate, there are relatively few published case studies of how this can play out through the lived experience of PPI in research; early findings highlight key issues around access to knowledge, resources, and interpersonal respect. This article describes the findings of a case study of PPI within a study *about* PPI in research.

**Methods**

The aim of the study was to look at how the PPI representatives’ inputs had developed over time, key challenges and changes, and lessons learned. We used realist evaluation and normalisation process theory to frame and analyse the data, which was drawn from project documentation, minutes of meetings and workshops, field notes and observations made by PPI representatives and researchers; documented feedback after meetings and activities; and the structured feedback from two formal reflective meetings.

**Results**

Key findings included the need for named contacts who support, integrate and work with PPI contributors and researchers, to ensure partnership working is encouraged and supported to be as effective as possible. A structure for partnership working enabled this to be enacted systematically across all settings. Some individual tensions were nonetheless identified around different roles, with possible implications for clarifying expectations and deepening understandings of the different types of PPI contribution and of their importance. Even in a team with research expertise in PPI, the data showed that there were different phases and challenges to ‘normalising’ the PPI input to the project. Mutual commitment and flexibility, embedded through relationships across the team, led to inclusion and collaboration.

**Conclusion**

Work on developing relationships and teambuilding are as important for enabling partnership between PPI representatives and researchers as more practical components such as funding and information sharing. Early explicit exploration of the different roles and their contributions may assist effective participation and satisfaction.

## Background

Major national and regional research funding programmes in the U.K. and other developed countries now expect that genuine (not “tick-box”) patient and public involvement (PPI) is prioritised and resourced within studies. The rationale and value of partnership working between researchers and PPI representatives is highlighted in the recent ‘Extra Mile’ report commissioned by the U.K. Chief Medical Officer [1], which includes a plea that “*Evidence of what works is* (made) *accessible so that others can put it into practice”.* The policy momentum for PPI has been building for almost two decades [2], but evidence of how and why PPI can improve research outputs is still emerging,’ [3]. For this reason, the National Institute for Health Research funded the RAPPORT study, [4] whose aim was to *‘To determine the types of PPI in funded research, describe key processes, analyse the contextual and temporal dynamics of PPI, and explore the experience of PPI in research for all those involved’”.* The main methods and findings of this study are reported elsewhere, but for the purposes of the RAPPORT study, the key characteristics of effective PPI within individual research studies were operationalised as evidence indicating:

- *“a shared understanding of the moral and methodological purposes of PPI*
- *a key individual co-ordinating PPI*
- *ensuring diversity* (of PPI representation and inputs)
- *a research team positive about PPI input and fully engaged with it, based on*
- *relationships that were established and maintained over time, and*
- *PPI being evaluated by a proactive and systematic approach.”* ^4^

Patient and public involvement is a process influenced by many factors. To address this the RAPPORT study used realist evaluation (RE) [5]. This form of theory-driven evaluation, aims to advance understanding of why complex interventions, such as PPI work, how, for whom, in what context and to what extent, and also to explain the situations when a programme fails or has only limited effect.. This approach enabled us to address in depth how different theories around how PPI works as an integral part of the research process. We were also interested in whether widespread prioritising of PPI in U.K. policies has led to it becoming embedded as normal practice within U.K. research. We used Normalisation Process Theory [6] (NPT) to structure our examination of how PPI may become routine alongside other key components of research,.

The RAPPORT study selected 22 ‘case studies’ of currently funded research projects adopted on the United Kingdom Clinical Research Network portfolio, and analysed the different approaches and extent of PPI. This enabled us to include a range of studies by research approach, focus, location and involvement of PPI representatives.

From the outset its study team was aiming for partnership between PPI representatives and researchers. We became increasingly conscious that there was a valuable case study about how this PPI/researcher partnership was negotiated, introduced and reviewed as part of the report’s findings and learning. The need to learn from one’s own experiences and the value of the subjective voice is recognised in social science approaches [7]. This paper therefore analyses on the data collected about the RAPPORT study’s own efforts to create and implement effective PPI, and the extent to which our own experiences reflect the conclusions of the RAPPORT study. We looked back at key ways in which the different PPI components developed over time, the challenges and barriers, changes made because of PPI inputs, and evidence of outputs and lessons learned. We make additional recommendations for others based on this analysis.

One of the complexities of this area is finding appropriate words to convey the varying roles and related values which are enacted under the overarching construct of PPI [8]. In the RAPPORT report, we used ‘PPI representatives’ to cover all PPI input, and this article uses the same term, though specific different roles are described in the findings.

## Methods

The evaluation of PPI within RAPPORT was guided by participative approaches [9]. As for the main study we used NPT as an explanatory theory to critically evaluate the extent of our own ‘normalisation’ of PPI within RAPPORT and so we used the four constructs of NPT as a framework for coding the different types of data and to inform analysis of the extent of and ways in which PPI was ‘normalised’ in the research projects, as well as what helped or hindered this. These four constructs are: a sense of coherence (sense making/‘how does this fit?’); participation (making the effort/initiating a change process); collective action (doing the work to make the process effective); and reflective monitoring (appraising the impact of changing approaches and implementation).

Data used for the analysis included project documentation (such as protocol version development and personal inputs to this); contemporary minutes of meetings and workshops (including reference group discussions, field notes and contributions of the PPI representatives and researchers); feedback after meetings and activities (including feedback by co-researchers after they had conducted interviews); resources offered to support PPI; and the structured feedback of two formal, independently-run, reflective meetings.^1^

Using NVivo software, initial coding was conducted by EM, JK, AH, and PW, and then collated into a first draft of detailed findings by AH. This was then iteratively discussed with all authors until consensus was achieved, to develop key themes that had supported ‘normalisation’ of PPI (Table 2). We then structured our main findings around the two areas identified here as key, of ‘forms and processes’ and ‘experience and dynamics’ of PPI. All authors then contributed to revisions.

### Findings

The detail of the PPI input to RAPPORT is set out in the full report, here summarised in Table 1.

**Table 1:** Details of commitments of three main PPI groups in the RAPPORT protocol

PPI Role	Activity	Frequency of Activity (approx. hours)^a^
1. Co-applicants/ Co-researchers (3)	Contribute to research meeting Training (interviews/data analysis) Interviews Data Analysis meetings Presenting a INVOLVE conference Other activities*: research idea/design, data analysis (reading transcripts), commenting on documents, final report, writing dissemination materials, preparation for meetings/conferences.	Every 3 months 11 meetings (each 2–3 h) 2 day conference 17 interviews (each 30–60 mins) plus all related work outlined here
2. Advisory Group Members (6 PPI representatives in total)	Reading papers before meeting, commenting during meeting.	Every 6 months
3. Patient Groups (reference) (2 - IDD and Cystic Fibrosis) Groups	Comment on documentation Comment on findings	2 meetings

^a^Other activities such as reading interview transcripts, reading meeting papers, conference preparation were additional hours. Accurate records of hours for all the PPI activities were not kept as part of this project but for example one co-researcher spent approximately 2 h commenting on an interview guide for PPI representatives, 8 versions were drafted for a dissemination article for a magazine, 50 detailed comments were made on the final report and over 19 h were spent writing up themes extracted from the interviews (not including time taken to read the transcripts). The total hours of PPI activity as co-researchers is estimated to be “100 s of hours” over the course of the study. The Table illustrates the three different PPI roles have different expectations and very different time commitments

### Forms and processes

The forms and processes underpinning RAPPORT shaped the specific enactment of PPI in practice. The original research idea and design was co-produced between researchers and members of two existing PPI groups – the University of Hertfordshire (UH) PIR group,^2^ and the Norfolk and Suffolk PPIRes group^3^ local to the University of East Anglia (UEA) [10]. It was agreed that there should be three different levels of PPI input – (1) PPI representatives as members of the research team (here, both as co-applicants and co-researchers); (2) PPI representatives as members of a research advisory group; and (3) PPI representatives as members of reference groups (as members of patient groups working on specific research topics, for example, setting up meetings with a local Intellectual and Developmental Disabilities (IDD) group and a cystic fibrosis group. The PPI patient groups were consulted independently of other within-project PPI groups by the team members at UH who fed back their contributions to the wider research team. The PPI members of the research team and the PPI advisory group members met regularly at advisory group meetings. The PPI representatives on the research team and advisory group were experienced in PPI activities and to encourage peer support encouraged efforts to recruit in pairs from support organisations.

This partnership approach could here be based on a wide range of pre-existing relationships. The project was hosted and led by UHwhose PIR group provided PPI representatives to meet co-applicant and co-researcher roles, while the UEA-linked PPIRes team provided input to the advisory group. The Advisory Group also included a chair who was a PPI representative, a PPI representative from the Royal College of General Practitioners (RCGP) and representatives linked to other reference groups; National Cancer Research Network (NCRN) and Mental Health Research Network (MHRN) and a Learning Disability Research and Development Manager. PPI activities were funded.

Arrangements were made for all PPI representatives to be included in induction and ongoing training and support, a named lead contact at each site, and their choice of communication (email, telephone, hard copy). Most PPI representatives were experienced in PPI through working on other research projects and policy and charity advisory panels and most also received training within their organisations. All were provided with project induction and offered ongoing support reflecting their roles and expressed needs and requirements. Further training and support around specific tasks and roles were provided by the researchers, particularly for co-researchers undertaking data collection and contributing to data analyses. Ongoing training (with support) sought to negotiate ways to value the strengths and abilities of the lay perspectives of the co-researchers, not with the intention of training PPI representatives as professional qualitative researchers, but to support their unique insights in relation to the aims and research principles of the project.

The co-researchers received ongoing training and support, had honorary contracts, and payment for their involvement. All team meetings included systematic opportunities to brief PPI members of the team before and debrief after all meetings with named lead contacts for different activities. Systems were set in place that addressed explicit requests for feedback about processes and other activities; and supported the use of reflective diaries, feedback sheets and other structured, recurrent input opportunities. For example, PPI was an integral agenda item for all meetings, as the research team.had an overall aim to have consistent PPI throughout, including involving the PPI representatives in co-applicant/co-researcher roles, in study design and delivery. Given this intention, and the funding for PPI made explicit from the outset, it may be less surprising that the aim of routinely incorporating PPI into the research process, was fulfilled. All PPI representatives remained with RAPPORT throughout, and the only ‘break in service’ was due to one individual’s period of ill health.

### Experiences and dynamics

The data shows that, over time, the processes used to enable PPI were flexibly adjusted to reflect the experiences of project members and partners. For example, both PPI representatives and researchers encountered difficulties in maintaining ‘reflective diaries’, who gave greater priority to face to face conversations as less time consuming and more likely to be effective in dealing with any difficulties. All PPI representatives and researchers expressed growing confidence in all roles over time. Evidence for these strengthening relationships, was seen in how PPI demonstrably shaped the research process, for example, as documents altered in the light of PPI inputs and in data analysis. ‘What worked’ was stated to include: the multiple modes of PPI input, which allowed a PPI ‘voice’ to be heard across different settings; the explicit support within project structures such as having named team member to links with the PPI representatives; mutual commitment to building relationships; and whole-team responsiveness to concerns. In NPT terms, the view of the team was coherent with those of PPI representatives, all having worked in these ways before and having been motivated to seek research funding on the topic of PPI itself and to work in PPI-consistent ways. Evidence of ‘collective action’ (the third phase of normalisation) was shown by the research team’s records of rapid responses and actions taken following suggestions made by PPI representatives.

However there were some initial tensions, mainly around the different PPI roles of co-applicants/co-researchers and of research advisory group members. Practical challenges were experienced ensuring inclusion (explaining jargon, talk-time in committees, and help with technical aspects). Our evaluation findings showed variation between views of PPI representatives, some (a minority) expressing concerns, while most understood and expressed themselves content with the scope and remit of their roles. Data shows questions about scope and involvement as decreasing as the project went on, and were never experienced as too serious, as one PPI representative said “*As a co-researcher who was closely involved throughout several years of highly co-operative working, I concluded that it was remarkable how few tensions there were [within the project team]”.*

Nevertheless, an independently-facilitated event enabling the research team to reflect on the PPI process (held towards the end of the project) revealed a complex undertow of perspectives which highlight many of the issues identified by all parties (summarised in Table 2). A second reflective meeting for the Advisory Group members (internally-facilitated) was then held to review these.

**Table 2:** Key themes in data analysis

Coded theme	Dimensions of the issue and reflective questions
Choice and limitations	The problem of different roles, scope and resourcing *Who decides? Can PPI representatives change/increase their input if they want to?*
Accessibility	Literacy (health, educational, cognitive); mobility; distance; digital. *What are ‘reasonable’ adjustments? Can we ever be inclusive enough?*
Diversity	Socio-demographics of the ‘available and willing’ are not reflective of needs for some studies – different models can overcome this but *how can we ensure diversity of views?* The ‘professional’ PPI representative – conflict between growing their expertise over time and their need to maintain a ‘lay’ perspective
Team relationships	Important enabler of PPI input and maximising contribution. Can become obligated/collusive. Need to avoid dependency.
Skills	Interpersonal skills really important especially for the main PPI link. Also understanding of group dynamics. Both researchers and PPI representatives need to be open to new ideas and learning.
Attitudes	Respect, professionalism, honesty in all concerned (or involved)
Infrastructure	The right inputs at all stages Gaining and retaining funding Gaining and retaining skills over time Using networks
Bureaucratic issues	‘Employment’ or ‘volunteer’ status Pay/tax/human resource requirements./consequences for benefits
Boundary issues	Risks where personal needs and priorities dominate the PPI input Pushback – not accepting limits of role or resources
Impact	*How do we define and measure impact?!*

The conclusions of the RAPPORT team, as related to the four NPT stages, are shown in Table 3, also answering the research question about barriers and facilitators.

**Table 3:** Ingredients that facilitate good PPI

Coherence is assisted by: • All concerned having clear understanding and expectations of PPI • Having a process of selection for PPI representatives – so that people choose to join projects they are interested in/make sense to them • Role clarity being made important for all from start – though need some flexibility • Breadth/depth of PPI representatives understanding that different inputs have different aims • Avoiding heavy personal bias/advocacy towards particular issues and perspectives.
Buy in (participation) is assisted by: • Having named co-ordinator(s) • A moral commitment to PPI • Keeping PPI roles and expectations under review • Preparedness and flexibility for changing circumstances • ‘Don’t be politically correct’being clear about why and how you are working with and involving PPI representatives for specific research project and collaborator needs, rather than blindly following assumed ‘right’ ways of doing it • Making it fun by building relationships through some informal periods of contact • Clearing a space to talk about/reflect on how the PPI aspect is working for project/people.
Collective action is assisted by: • Promoting respect for difference by: role modelling, valuing, listening, inter-personal skills • Attending to relational/collaboration processes • Learning to balance the expectations of PPI representatives to be treated as colleagues with the different structural requirements of their volunteer status • Meeting people’s needs where appropriate • Allowing flexibility of methods of contact/communication • Identifying the right skill mix for different projects, recognising the needs of particular projects • Accepting different educational levels, IT competencies and cognitive levels •‘ People skills’ training for researchers (to foster good working relationships) • PPI representatives require basic training and support (induction/orientation) in order to make PPI representatives feel ‘safe’ • Facilitate end of project and next steps for PPI representatives • Accessibility: venue, timings • Expenses (means of paying volunteers simply through organisations without altering benefits, tax etc.) • Personalised recognition of situations e.g. tax • Planned resources and contingency funds for PPI • Staff support for PPI • An adequate core structure/funding (including for PPI in bid development)
Reflection: PPI strengthened by - • Talking to everyone at the outset about expectations and background • Keeping more regular records of PPI impacts • Reviewing PPI processes regularly throughout– what is/isn’t working well, what do we want to do to change/improve. • Broadening scoping outputs for all as in PPI representatives as partners in papers and conferences.

This study’s PPI was distinctive in providing diverse and extensive roles and responsibilities:– co-researchers adding new data; advisory group membership assuring accountability to the public, adjusting research processes, and re-considering routes for dissemination; while the patient groups assisting reflection on specific case studies. The latter group improved communications by anticipating accessibility challenges. We concluded that the ‘embedded’ or ‘intertwined’ PPI model (i.e. PPI running throughout the project over time, part of the ongoing team) can usefully be supplemented by a dynamic ‘out-reach and in-reach’ approach to recognise and address when different perspectives are needed.

For those PPI representatives who acted as co-researchers, they experienced this as involving a more complex development over time. For some this entailed acquiring new skills, building confidence to ‘cross over’ to the researcher role, and having their outputs (interview data) scrutinised by others. They deemed this role as rewarding: “*The pluses, was using your skills again … feeling like I still can use my brain and connected with people and identifying with them…”* (PPI Representative, co-researcher, reflective thoughts notes).

By contrast, some PPI representatives on the advisory group felt less engaged; “*I want to do more than you are asking me to do all the time. So I don’t feel I have really bonded, because I haven’t been to that many meetings, I have been on the periphery, which I think is what an advisory/steering group is…”* (PPI representative on advisory group). This view emerged despite pre-existing relationships and despite sustained and positive overall engagement. The analysis showed that this related to lack of acceptance by the PPI advisory group members of the boundaries of different roles as they came to experience them. This tension led to some of the suggestions identified in Table 2 (reviewed in the Discussion below). This role may well have specific valued for researchers in helping them meet the requirements of governance and funders, but for these PPI representatives was experienced as less personally engaging than that of a co-researcher. One of the researchers explained this as “*They (PPI representatives on advisory groups) are also often key in ensuring accountability to funders by anticipating, mediating and monitoring corrective action needed over the life of a project. However, experiences of* *how* *steering groups run may signal to PPI.. that they are just “business as usual” in terms of their routine and standardised consideration when a project is running well, and therefore it may not be obvious why they are so important”.*

Finally, for outputs, the PPI representatives were found to provide spontaneous contributions and novel resources. One developed an analytic tool for PPI representatives to reflect on their experience, using a scale and visual analogy of climbing a mountain, which was first used in the reflective meetings and has since been carried forward into other projects.^4^

In summary, the RAPPORT study team succeeded in normalising its PPI inputs by means of establishing structured approaches to assist coherence;; and worked on relationships through a sustained and comprehensive partnership approach to assist participation, by supporting the PPI representatives in their various roles, and making specific opportunities for additional inputs through the patient groups and co-researchers. This led to increasingly coherent activities and collective (rather than fragmented) actions. As one of the academic research team said in a meeting near the end:

*“Looking back it is interesting to reflect how positive RAPPORT PPI turned out in the end. Why was this? It can all be related to …*

- *Clarification and understanding of roles*
- *Tweaking roles to meet individual preferences*
- *Relationship building; “joining up the two centres,.. joining up the various groups,”*
- *A key person who was their main point of contact and was local to them*
- *Careful feedback on the actions taken in the study in response to our PPI [representatives] colleagues’ suggestions*
- *A successful project led to a feel good factor for all*
- *Effort to evaluate the PPI in RAPPORT*”.

## Discussion

This is one of the first papers on PPI which has documented, as an integral part of the research project, the process of involving people in different PPI roles on a specific project. The use of Normalisation Process Theory helped demonstrate the dynamic nature of PPI, and therefore the need to continue to test team assumptions about role, contribution and responsibilities over the lifetime of a project for whole-team engagement with and an active contribution from PPI.

The debates which emerged within this case study mirrored the findings of the RAPPORT study itself, which noted that personal presence did not always guarantee participation. “*The most common forum in our case studies was some form of study management committee. In many of these committees [PPI representatives] felt well supported and that their voice was heard, often primarily enabled by relational work … However, other PPI representatives reported feeling lost by language, procedures and lack of support, and unable to contribute effectively to these committees*” ^4.^ 5

There were aspects within this study that were clearly more about more general group dynamics than specific ways in which PPI embeds in a research project; both the emergence and reduction of challenges over time are typical of functional team development, as expressed in Tuckman’s adage that teams have to ‘form’ and ‘storm’ before they can ‘norm’ and perform’ [11].

While academic researchers on RAPPORT were clear that the accountability and governance function of PPI on the advisory group was well performed and had value, some PPI representatives perceived this as less valuable and less personally-fulfilling than a co-researcher role. The learning from this tension has led to the recommendations for more clarity in PPI roles at the start, and the need for awareness of and to address, potentially diverse intellectual and emotional reactions to different levels of embeddedness for PPI and researcher members within one project team.

There is also a systems question raised by this issue: how much value and impact does advisory group participation have? The U.K. Clinical Research Collaboration, having examined different perspectives on the role of PPI in advisory and oversight committees, stated firmly that PPI on advisory groups should continue, but recommended several ways to increase both its profile and support for this role [12]. The INVOLVE website clearly sets out the roles and values of different types of PPI input but using differing language for different roles and appears to convey co-researchers’ input is more embedded and impactful than that of advisers. How and how far advisory groups can contribute to ensuring accountability will depend on the specific needs of the project for accountability and the conduct of the project in practice and so the extent of any accountability challenges. All of this would condition the PPI representative’s role on the advisory group in practice advisory and be discussed with those to whom the role might be offered.

The RAPPORT study members intended from the outset that its structures should support control and choice for the PPI representatives. The issues that were subsequently experienced and reported echo a dynamic identified two decades ago by Beresford [13], who contrasted the consumerist and democratic roles of PPI in research so drawing attention to who drives the process: the public, seeking a voice to give research better value, to improve their own lives and to enable genuine participation or the establishment seeking societal permission for their actions yet retaining control of the inputs and outputs. The dynamics of power, within RAPPORT, despite a sophisticated PPI approach, experienced PPI representatives, PPI-allocated staff and resources, still required transparent acknowledgement of how the structures affected experienced PPI in the project to evidence and then to promote PPI as genuinely collective partnership in research production. The process of ensuring that PPI representatives and researchers work in a productive rather than tokenistic partnership was seen not to be guaranteed by specific structures and resources being put in place at the outset but to be dynamically reviewed and negotiated.

One limit of this study may be that it is based on a single case study, although its depth of data and analysis is a strength. The team was highly gendered, with no men on the co-researcher team and just one on the advisory group. The whole group had been involved over time with a study where key aspects of good practice in PPI were being explicitly discussed and debated, which may have reduced some of the known barriers to fully embedding PPI as integral to the study team, and also perhaps encouraged concerns about limits on PPI to be more expressed in more detail. That themes identified in this study had resonance with issues emerging in other studies supports the credibility of the findings. The specific detail provided here should enable some transferability to the work of others, and help focus further research that can test the key issues and recommendations suggested.

## Conclusions

The implementation work required to embed quality PPI relationships may often be delegated and can go unrecognised - but in this study was systematically examined by the use of a Normalisation Process Theory approach. The experience of this project identifies issues which reflect those of other reports [14] and of theoretical models of what makes PPI work in research. These identify the importance of an inclusive approach and of repeated efforts to ensure a strong and honest PPI voice, together maximising effective change and outputs [15]. Of all factors highlighted - structural, organizational, and relational - the most emphasis was seen to be placed on establishing relationships which can create safe interpersonal dynamics that enable PPI contributions to be freely made. Building these takes time, and opportunities need to continue to develop over the research study duration. Although in this case there was a designated role to facilitate relationships in each study project grouping, it became clear that - rather than relying solely on these facilitating roles to enable smooth PPI partnership - all concerned needed to share responsibility for working to share expectations and ongoing reflection. This highlighted the value of designing and investing in formative and summative, collective, reflective analyses of PPI contributions within and with project teams. Reflective analysis can and should be done in ways appropriate to the study itself, and independent facilitators here could be seen to play a useful role in facilitating teams to explore within and beyond their own working relationships, to help identify wider lessons. These indicate that enabling partnership between PPI representatives and researchers relies on valuing relationship-building at least as much as more practical components such as funding and information sharing.

## Abbreviations

IDD: Intellectual and developmental disabilities
PIRG: The University of Hertfordshire’s Public Involvement in Research Group
PPI: Patient and public involvement
PPIRes: The Norfolk and Suffolk NHS Patient and Public Involvement in Research Group
The RAPPORT study: ReseArch with Patient and Public invOlvement: a RealisT evaluation^4^
UEA: University of East Anglia
UH: University of Hertfordshire

## Footnotes

1 For further detail see the RAPPORT final report chapter 4.

2
http://www.herts.ac.uk/research/centres-and-groups/cripacc/public-involvement-in-research-group-pirg accessed 19 Dec 2016

3
http://nspccro.nihr.ac.uk/public-and-patient-involvement-in-research (PPIRes = PPI in Research)

4 The ALPS tool ‘Amander’s Levels of Participation Significance’ © Amander Wellings.

5 RAPPORT report, p.119-120.
